# Contribution of large genomic *BRCA1 *alterations to early-onset breast cancer selected for family history and tumour morphology: a report from The Breast Cancer Family Registry

**DOI:** 10.1186/bcr2822

**Published:** 2011-01-31

**Authors:** Letitia D Smith, Andrea A Tesoriero, Ee M Wong, Susan J Ramus, Frances P O'Malley, Anna Marie Mulligan, Mary Beth Terry, Ruby T Senie, Regina M Santella, Esther M John, Irene L Andrulis, Hilmi Ozcelik, Mary B Daly, Andrew K Godwin, Saundra S Buys, Stephen Fox, David E Goldgar, Graham G Giles, John L Hopper, Melissa C Southey

**Affiliations:** 1 Genetic Epidemiology Laboratory, Department of Pathology, University of Melbourne, Melbourne, Victoria 3010, Australia; 2 QIAGEN Pty. Ltd., Doncaster, Victoria 3108, Australia; 3 Gynaecological Cancer Research Laboratory, Institute for Women's Health, University College London, 74 Huntley Street, London, WC1E 6AU, UK; 4 Department of Pathology and Laboratory Medicine, Mt Sinai Hospital, 600 University Avenue, Toronto, ON, M5G 1X5, Canada; 5 Department of Pathobiology and Laboratory Medicine, University of Toronto, 1 King's College Circle, Toronto, ON, M5S 1A8, Canada; 6 St Michael's Hospital, 30 Bond Street, Toronto, ON, M5B 1W8, Canada; 7 Department of Epidemiology, Mailman School of Public Health, Columbia University, 722 West 168th Street, New York, NY 10032, USA; 8 Department of Environmental Health Sciences, Mailman School of Public Health, Columbia University, 722 West 168th Street, New York, NY 10032, USA; 9 Department of Epidemiology, Cancer Prevention Institute of California, 2201 Walnut Avenue, Fremont, CA 94538, USA; 10 Samuel Lunenfeld Research Institute, Mount Sinai Hospital, Department of Molecular Genetics, University of Toronto, Ontario Cancer Genetics Network, Cancer Care Ontario, 620 University Avenue, Toronto, ON, M5G 2C1, Canada; 11 Fox Chase Cancer Center, 333 Cottman Avenue, Philadelphia, PA 19111-2497, USA; 12 Huntsman Cancer Institute, University of Utah Health Sciences Center, Salt Lake City, UT 84112, USA; 13 Department of Pathology, Peter MacCallum Cancer Center, St Andrew's Place, East Melbourne, Victoria 3002, Australia; 14 Department of Dermatology, University of Utah School of Medicine, Salt Lake City, UT 84112, USA; 15 Cancer Epidemiology Centre, The Cancer Council Victoria, Rathdowne Street, Carlton 3052, Australia; 16 Centre for Molecular, Environmental, Genetic and Analytic Epidemiology, University of Melbourne, Melbourne, Victoria 3010, Australia; 17 Department of Pathology and Laboratory Medicine, University of Kansas Medical Center, 3901 Rainbow Blvd, Kansas City, KS 66160, USA

## Abstract

**Introduction:**

Selecting women affected with breast cancer who are most likely to carry a germline mutation in *BRCA1 *and applying the most appropriate test methodology remains challenging for cancer genetics services. We sought to test the value of selecting women for *BRCA1 *mutation testing on the basis of family history and/or breast tumour morphology criteria as well as the value of testing for large genomic alterations in *BRCA1*.

**Methods:**

We studied women participating in the Breast Cancer Family Registry (BCFR), recruited via population-based sampling, who had been diagnosed with breast cancer before the age of 40 years who had a strong family history of breast or ovarian cancer (*n *= 187) and/or a first primary breast tumour with morphological features consistent with carrying a *BRCA1 *germline mutation (*n *= 133; 37 met both criteria). An additional 184 women diagnosed before the age of 40 years who had a strong family history of breast or ovarian cancer and who were not known to carry a germline *BRCA1 *mutation were selected from among women who had been recruited into the BCFR from clinical genetics services. These 467 women had been screened for *BRCA1 *germline mutations, and we expanded this testing to include a screen for large genomic *BRCA1 *alterations using Multiplex Ligation-dependent Probe Amplification.

**Results:**

Twelve large genomic *BRCA1 *alterations were identified, including 10 (4%) of the 283 women selected from among the population-based sample. In total, 18 (12%), 18 (19%) and 16 (43%) *BRCA1 *mutations were identified in the population-based groups selected on the basis of family history only (*n *= 150), the group selected on the basis of tumour morphology only (*n *= 96) and meeting both criteria (*n *= 37), respectively.

**Conclusions:**

Large genomic alterations accounted for 19% of all *BRCA1 *mutations identified. This study emphasises the value of combining information about family history, age at diagnosis and tumour morphology when selecting women for germline *BRCA1 *mutation testing as well as including a screen for large genomic alterations.

## Introduction

For many women with a personal and/or family history of breast cancer, knowing that they carry a germline mutation in a breast cancer predisposition gene can be informative for their clinical management and that of family members. However, mutations in currently known breast cancer predisposition genes are very rare, and the majority of women who undergo genetic testing for these genes are not found to be carriers of mutations (identifiable by current molecular methods).

It remains challenging but necessary for clinical genetics services, and in some settings, insurers, to select women most likely to carry a germline mutation in *BRCA1 *and *BRCA2 *for genetic testing. Most tools used for estimating a woman's risk of carrying a mutation in one of these genes are predominantly based on family history. Even when accurately reported, well collected and verified, family history is often not predictive of carrier status unless it is extreme, as family history is not highly sensitive or specific to mutation status [1]. Recent and current work have incorporated some details of the associated breast cancer pathology into risk models, but this effort has essentially been restricted to immunohistochemical data such as estrogen receptor (ER), progesterone receptor (PR), HER2 and cytokeratin (CK5/6, CK14) status [2-4].

It has been widely reported that some breast tumour morphological features are associated with carrying a *BRCA1 *mutation and that consideration of a few of these features can identify almost all *BRCA1 *germline mutation carriers among women with early-onset breast cancer without taking into account family history [5-7].

The methodological approaches applied to identify *BRCA1 *mutations also vary between clinical service laboratories and can have an impact on the proportion of women identified as carrying a mutation [8]. Most currently applied methods are based on polymerase chain reaction (PCR) assays and have moderate to high specificity and sensitivity for identifying mutations involving one or a few nucleotides within defined, predominantly exonic regions. Analytical methods for interpreting these nucleotide alterations are continuing to improve [9,10]. However, short-fragment, PCR-based analyses are usually insensitive to the detection of large genomic alterations, and unless these specific tests are applied such mutations are likely to be missed in a routine short-fragment PCR-based screening protocol.

The frequency of large genomic alterations varies between populations, and this has become more apparent as methods to detect such mutations have become more robust and available [11]. Ticha *et al*. [12] reported that 12.3% of all *BRCA1 *mutations identified in the Czech population are large genomic rearrangements. Similarly, Agata *et al*. [13] reported that they could represent up to one-third of *BRCA1 *mutations identified in the Italian population. Reports from the Netherlands have demonstrated that these types of mutations can account for 27% to 36% of all *BRCA1 *mutations in the Dutch population [14,15]. In addition to the effect of founder mutations, some regions of *BRCA1 *seem to be implicated more frequently in genomic alterations, such as the exon 1A-2 region [16]. The value of large genomic rearrangement testing for many clinical genetics services remains unclear.

We sought to further investigate the value of using family history and tumour morphologic features in selecting women for *BRCA1 *mutation testing. We applied two criteria (one for family history and one for tumour morphological features) to select women participating in the Breast Cancer Family Registry (BCFR) who had already undergone extensive *BRCA1 *mutation screening [17]. This testing had not, however, included routine testing for large genomic alterations in the *BRCA1 *region, so we applied Multiplex Ligation-dependent Probe Amplification (MLPA; MRC-Holland, Amsterdam, the Netherlands) testing to determine the proportion of *BRCA1 *mutations in the selected women that could be attributed to mutations of this type.

## Materials and methods

### The Breast Cancer Family Registry

The BCFR was established in 1995 as an international collaborative resource to facilitate research into the genetic and environmental causes of breast cancer. The BCFR has collected detailed epidemiological data, family history information and biospecimens from over 13,000 families [18]. Relevant to this study are the incidence of breast cancer cases and their relatives ascertained through population-based cancer registries (population-based case families) and families with strong cancer histories of breast or ovarian cancer identified through cancer family clinics and community outreach (clinic-based families). Population-based families were recruited in the San Francisco Bay Area, northern California, USA; the Province of Ontario, Canada; and Melbourne and Sydney, Australia. Clinic-based families were recruited in Philadelphia, New York City and Utah, USA; the Province of Ontario, Canada; and Melbourne and Sydney, Australia. A proband is defined as the index case (identified from the relevant cancer registry) in population-based families and as the youngest affected participating female member of clinic-based families. All sites used standardised questionnaires and protocols to collect family history information, epidemiological and clinical data, and biological specimens, with a strong emphasis on quality control measures throughout the collection, processing and storing of data and samples [17,18]. This study was approved by the Human Research Ethics Committee of The University of Melbourne.

### *BRCA1 *and *BRCA2 *mutation testing

BCFR participants have undergone extensive testing for *BRCA1 *and *BRCA2 *mutations using techniques described previously [8,17,19], including two-dimensional gel scanning, denaturing high-performance liquid chromatography, enzymatic mutation detection, single-strand conformation polymorphism analysis and the protein truncation test. Direct gene sequencing of both genes has also been carried out by individual laboratories and Myriad Genetics (Salt Lake City, UT, USA) [8,17,19]. Mutation testing in other genes, such as *ATM*, *TP53 *and *CHEK2*, has also been performed and reported elsewhere [20-22]. The criteria used by the BCFR for defining deleterious mutations are the same as those used by the Breast Cancer Information Core [23] and Myriad Genetics. No screening was applied that specifically tested for large genomic alterations in the *BRCA1 *gene. Some carriers of *BRCA1 *duplication exon 13 were identified by specific PCR-based testing for the breakpoints [19,24] and via RNA-based protein truncation testing [8].

### Selection criteria

#### Women recruited through population-based sampling

##### Family history criteria

We selected case probands who were diagnosed with breast cancer before age 40 years and had two or more first- or second-degree relatives with breast or ovarian cancer.

##### Tumour morphology criteria

We selected case probands whose tumour morphology was consistent with carrying a *BRCA1 *mutation [7]. Case probands from the Australian BCFR were selected if their breast tumours had five or more of the following morphological features: (1) mitotic index >50/10 high-power fields (HPF), (2) malignant nuclear grade, (3) little or no tubule formation, (4) a trabecular growth pattern, (5) pushing margins (>50%), (6) a circumscribed growth pattern, (7) a syncytial growth pattern, (8) necrosis and (9) moderate or intense lymphocytic infiltrate [5,7]. From the Northern California BCFR, we first selected all breast cancers that had been scored as medullary or atypical medullary as their primary or secondary histological type and then reviewed the histological slides of these cases to identify those that met the criteria described above [7]. From the Ontario BCFR, we selected cases that had five or more of the following features: (1) mitotic index >50/10 HPF, (2) malignant nuclear grade, (3) little or no tubule formation, (4) a syncytial growth pattern, (5) circumscribed borders, (6) a moderate intense lymphocytic infiltrate and (7) necrosis. The selection of case probands from population-based registries was done without regard to their known *BRCA1 *or *BRCA2 *mutation status.

#### Women enrolled through clinic-based recruitment

We selected the youngest affected member of clinic-based families for this study if they had been diagnosed with breast cancer before age 40 years and had two or more first- or second-degree relatives with breast and/or ovarian cancer. Probands were excluded if they were already known to carry a *BRCA1 *or *BRCA2 *deleterious mutation.

### *BRCA1* large genomic alteration testing by Multiplex Ligation-dependent Probe Amplification

Testing for large genomic alterations was performed by MLPA using the SALSA MLPA Kit P002B BRCA1 (MRC-Holland) as described by Schouten *et al*. [25]. Quantities of 50 to 100 ng per reaction of DNA extracted from Guthrie card blood spots, peripheral whole blood or a lymphoblastoid cell line were used. All reactions were performed in duplicate on a Corbett Palm-Cycler, Corbett Life Science, Mortlake, NSW, Australia, and PCR fragments were analysed on an ABI 3730 DNA Analyser (Applied Biosystems, Foster City, CA, USA).

Analysis of the fragment peak areas and visual examination of the MLPA histograms were performed to identify large genomic alterations [26]. A normalised value of 1.0 represents the detection of both exonic alleles (that is, no alteration), a value ≤0.65 is the threshold suggestive of loss of one exonic allele (that is, deletion) and a value ≥1.3 suggests the gain of one or more exonic alleles (that is, duplication). All cases with normalised values ≤0.65 or ≥1.3 were repeated in an independent molecular analysis. Large alterations were verified using the SALSA MLPA Kit P087 BRCA1 (MRC-Holland). For P087 analysis, cases were compared with *BRCA1 *mutation-negative controls. Cases that had MLPA analysis suggestive of a single exon loss or gain were sequenced to confirm that probe hybridization was not being disrupted by genetic variation in the MLPA probe hybridization regions.

## Results

### Screening of the population-based probands

Two hundred eighty-three probands from population-based families were selected for this study. Of these probands, 150 met the family history only criteria, 96 met the tumour morphology only criteria and 37 fulfilled both criteria. *BRCA1 *and *BRCA2 *mutation testing had already identified 15 *BRCA1 *mutation carriers in the family history only group (15 of 150, or 10%), 14 *BRCA1 *mutation carriers in the morphology only group (14 of 96, or 15%) and 12 *BRCA1 *mutation carriers in the group who met both criteria (12 of 37, or 32%). There were 17 *BRCA2 *mutation carriers who had been identified in the family history only group (17 of 150, or 11%), one *BRCA2 *mutation carrier in the morphology only group (1 of 96, or 1%) and four met both criteria (4 of 37, or 11%) (see Table 1).

**Table 1:** Women recruited into BCFRs on the basis of population-based sampling who met the family history and tumour morphology criteria of this study^a^

Mutation status of proband	**Family history only, 44/53/53**^**b **^**(*n *= 150)**	**Family history**^**c**^**, 63/57/67**^**b **^**(*n *= 187)**	**Tumour morphology only, 80/14/2**^**b **^**(*n *= 96)**	**Tumour morphology**^**d**^**, 99/18/16**^**b **^**(*n *= 133)**	**Both family history and tumour morphology, 19/4/14**^**b **^**(*n *= 37)**	**Study total, 143/71/69**^**b **^**(*n *= 283)**
No identified mutation, *n*	110	127	77	94	17	204
Identified mutation, *n*	40	60	19	39	20	79
*BRCA1 *LGA, *n*	3	7	3	7	4	10
	1/1/1^b^	3/3/1^b^	3/0/0^b^	5/2/0^b^	2/2/0^b^	6/3/1^b^
Other *BRCA1 *mutation, *n*	15	27	15^e^	27^e^	12	42
	1/8/6^b^	8/9/10^b^	14/1/0^b^	21/2/4^b^	7/1/4^b^	22/10/10^b^
*BRCA2 *mutation, *n*	17	21	1	5	4	22
*ATM, CHEK2, TP53 *mutations, *n*	5	5	0	0	0	5
*BRCA1 *mutations, %	12	18	19	26	43	18
*BRCA1 *LGA, %	17	21	17	21	25	19

^a^BCFR, Breast Cancer Family Registry; LGA, large genomic alteration; *ATM*, ataxia telangiectasia mutated gene; *CHEK2*, CHK2 checkpoint homolog gene; *TP53*, tumour protein 53 gene; ^b^data divided by BCFR (Australia/northern California/Ontario BCFRs); ^c^including women who also met the tumour morphology criteria; ^d^including women who also met the family history criteria; ^e^including *BRCA1 *4362delG identified by using Multiplex Ligation-dependent Probe Amplification during this study.

Table 1 shows that MLPA analysis identified eight case probands as carriers of large *BRCA1 *genomic alterations. Two additional case probands were known to have a *BRCA1 *exon 13 duplication that had been identified by previous testing, and these are included in Table 1. The *BRCA1 *large genomic alterations included five single-exon deletions (exon 5, exon 17 and exon 20) or duplications (exon 13) and five alterations involving the deletion of multiple exons (exon 1A-2, exon 1A-17, exon 1A-23 and exon 1A-24). An additional proband was found to have an MLPA profile consistent with an exon 13 deletion. However, Sanger sequencing of the exon revealed a 1-bp deletion adjacent to the ligation site of the P002 exon 13 probes. This mutation was characterised as *BRCA1 *4362delG and is reported in the "Other *BRCA1 *mutation" row in Table 1. The detection of this mutation had been missed by prior two-dimensional gel electrophoresis testing.

By combining the data from the screen for large genomic alterations with the mutation information that had been generated prior to this study using routine screening methods [17], we identified 18 probands (12%) in the family history only group carrying a deleterious *BRCA1 *mutation (3 probands, 2% carried large genomic alterations), 18 probands (19%) in the tumour morphology only group carrying a *BRCA1 *mutation (3 probands, 3% carried large genomic alterations) and 16 probands (43%) in the group meeting both criteria and carrying a *BRCA1 *mutation (4 probands, 11% carried large genomic alterations) (Table 1). Overall, 10 (19%) of the 52 *BRCA1 *mutations found in these women were large genomic alterations.

As there were some differences in the methods used to select cases that qualified for inclusion in the morphology group across the population-based BCFRs (see Materials and methods), the outcomes of the *BRCA1 *testing are detailed both as overall findings and for each BCFR individually (population-based samples) in Table 1.

### Screening of probands recruited from clinical services

There were 184 case probands from the BCFR clinic recruitment participants that met our study criteria (excluding *BRCA1 *and *BRCA2 *mutation-carrying probands). MLPA screening identified a *BRCA1 *exon 22 deletion (reported previously) and a *BRCA1 *exons 14-20 deletion. Histological sections stained with haematoxylin and eosin were available for review from the breast cancer carrying the *BRCA1 *exon 22 deletion, and this section was found to meet the tumour morphology criteria applied to the population-based probands in this study (Table 2).

**Table 2:** Details of *BRCA1 *large genomic alterations identified in this study

Sample	Selection criteria (*n*)	Large genomic *BRCA1 *alteration	Proband age at breast cancer diagnosis, yr	**Tumour morphology score**^**a**^	Family history (age at diagnosis, yr)
Population-based	Family history (150)	Deletion exon 5	38 and 44	3	Breast, sister (42)
					Ovarian, paternal aunt (44)
					Ovarian, paternal grandmother (60)
		Deletion exon 1A-24	39 and 49	3	Breast, sister (53)
					Ovarian, sister (40)
					Ovarian, sister (50)
		Duplication exon 13^b^	39 and 39	3	Breast, sister (33)
					Breast, mother (49)
					Breast, maternal grandmother (62)
					Breast, paternal grandmother (49)
	Tumour morphology (96)	Deletion exon 20	31 and 35	7	Breast, paternal grandmother (n.d.)
		Deletion exon 1A-23^c^	33	5	Breast, paternal aunt (71)
		Duplication exon 13^b^	36	9	Breast, mother (50)
					Ovarian, mother (68)
					Ovarian, paternal aunt (55)
	Family history and tumour morphology (37)	Deletion exon 17	31	7	Breast, maternal aunt (34)
					Ovarian, maternal aunt (35)
		Deletion exon 1A-2^c^	29	7	Breast, maternal aunt (30)
					Breast, paternal aunt (52)
					Ovarian, mother (56)
		Deletion exon 1A-17	36 and 38	8	Breast, mother (53)
					Ovarian, mother (62)
					Breast, maternal grandmother (84)
					Ovarian, maternal grandmother (n.d.)
		Deletion exon 1A-23	34	7	Breast, mother (28)
					Breast, maternal grandmother (45)
Clinic-based	Family history (184)	Deletion exon 22	35	7	Breast, paternal aunt (23 and 24)
					Ovarian, paternal aunt (49)
		Deletion exons 14-20	36	n.d.	Breast, sister (35)
					Breast, mother (43)
					Ovarian, maternal grandmother (n.d.)
Total	467	12			

^a^Tumour morphology score is the total number of the following features that each breast cancer was recorded to display: 1, mitotic index >50/10 high-power fields; 2, malignant nuclear grade; 3, little or no tubule formation; 4, a trabecular growth pattern; 5, pushing margins (>50%); 6, circumscribed growth pattern; 7, a syncytial growth pattern; 8, necrosis; and 9, moderate or intense lymphocytic infiltrate; ^b^*BRCA1 *exon 13 duplications were identified prior to this study; ^c^described by Smith *et al*. [26]; n.d., age at diagnosis unknown (no data).

Table 2 shows the age at breast cancer diagnosis, the details of the tumour morphology and the family history details of the young women found to carry a large genomic alteration in *BRCA1 *from both the population-based and clinic-recruited families participating in the BCFR. All 12 large genomic alterations in *BRCA1 *reported in this study were verified using the P087 MLPA Kit.

## Discussion

Knowing that a woman with breast cancer carries a germline *BRCA1 *mutation informs her clinical management and that of her relatives. However, it remains challenging for clinical genetics services to select the women most likely to carry a germline mutation in *BRCA1 *for genetic testing. Most tools used for estimating a woman's probability of carrying a *BRCA1 *mutation have been based predominantly on family history. Even when accurately reported, well-collected and verified, is often unhelpful, except when the history is extreme. That is family history is not highly sensitive or specific to *BRCA1 *mutation status.

We have investigated the relative strengths of using family history and tumour morphology, as well as both criteria together, to select the women with early onset breast cancer most likely to carry *BRCA1 *mutations (including large genomic alterations) by using the BCFR [18]. This resource provided many strengths for the study: (1) it provided a large sample size, with 467 women meeting one or more of the study criteria; (2) significant germline mutation testing had already been performed for *BRCA1 *and *BRCA2 *[17] using validated methods [8]; (3) family history of breast or ovarian cancer had been collected from each case proband using a standardised, validated instrument (ensuring higher-quality data than would likely have been collected from a similar number of women in a clinical setting) [18]; and (4) tumour morphology had been reviewed for a proportion of the breast cancers using a standardised, validated review form [7,27].

Prior to this study, 149 case probands (4%) who had been recruited into the BCFR via population-based sampling had been found to carry a *BRCA1 *mutation [17]. This is consistent with the frequencies reported in other studies utilising this and similar population-based samplings [28-30].

Using family history data collected in a standardised fashion by the BCFR, we identified 187 women recruited via population-based sampling who had a family history at least as strong as two first- or second-degree relatives with breast or ovarian cancer. Genetic testing of these women identified 60 (32%) with a mutation in a breast cancer susceptibility gene, 34 (18%) in *BRCA1*.

Using tumour morphology data collected by a standard pathology review of many of the breast cancers arising in women participating in the Australian and Ontario BCFRs, and by performing an extended review of the medullary and atypical medullary type breast cancers in the northern California BCFR (see Materials and methods), we identified 133 women whose tumour morphology met our selection criteria and thus had many of the morphological features consistent with being a *BRCA1 *mutation carrier. Of these, 39 (29%) have now been found to carry a germline mutation in a breast cancer susceptibility gene, 34 (26%) in *BRCA1*.

The women who met both criteria composed the group most enriched for *BRCA1 *mutation carriers (43%). The proportion of large genomic alterations in the total number of mutations identified was also slightly higher in the women who met both the family history and tumour morphology criteria (25%) compared with the proportion in women who met only one of these criteria (both 20%).

Similar work involving large genomic alterations in *BRCA2 *could also be beneficial, but it is likely to have less impact given current data which suggests there is a broader morphological phenotype of breast cancers carrying these mutations and the extreme rarity of these mutations in *BRCA2 *[31].

## Conclusions

These results highlight the value of incorporating information on family history and tumour morphology when selecting women with the highest chance of carrying a mutation in *BRCA1 *for mutation testing. This study also shows that testing for large genomic alterations is of value in this setting because 10 (19%) of the 52 mutation carriers indentified in the groups of women selected for this study carried large genomic alterations that were identifiable by using MLPA.

## Abbreviations

BCFR: Breast Cancer Family Registry; MLPA: Multiplex Ligation-dependent Probe Amplification.

## Competing interests

The authors declare that they have no competing interests.

## Authors' contributions

LDS, AAT and EMW conducted molecular analyses and contributed to data interpretation and manuscript preparation. SJR and HO contributed expert molecular assistance and contributed to the manuscript preparation. FPO, SF and AMM contributed morphological data and pathology expertise and contributed to the manuscript preparation. MBT, RTS, RMS, EMJ, ILA, MBD, AKG, SSB, DEG, GGG and JLH contributed data and DNA from participants of the BCFR and contributed to the manuscript preparation. MCS led this study, including its conception and design, acquisition of data and biospecimens, analysis, interpretation of data and preparation of the manuscript.
